# Proton-transfer rate constants for the determination of organic indoor air pollutants by online mass spectrometry†

**DOI:** 10.1039/d3ra01705b

**Published:** 2023-06-13

**Authors:** Tunga Salthammer, Uwe Hohm, Marcel Stahn, Stefan Grimme

**Affiliations:** a Fraunhofer WKI, Department of Material Analysis and Indoor Chemistry 38108 Braunschweig Germany tunga.salthammer@wki.fraunhofer.de; b Institute of Physical and Theoretical Chemistry, University of Braunschweig – Institute of Technology 38106 Braunschweig Germany; c Mulliken Center for Theoretical Chemistry, Institute for Physical and Theoretical Chemistry, University of Bonn 53115 Bonn Germany

## Abstract

Proton transfer reaction mass spectrometry (PTR-MS) has become an indispensable analytical tool for indoor related sciences. With high-resolution techniques not only is the online monitoring of the selected ions in the gas phase possible, but also, with some limitations, the identification of substance mixtures without chromatographic separation. The quantification is carried out with the help of kinetic laws, which require knowledge of the conditions in the reaction chamber, the reduced ion moblilities and the reaction rate constant *k*_PT_ under these conditions. Ion–dipole collision theory can be used to calculate *k*_PT_. One approach is an extension of Langevin's equation and is known as average dipole orientation (ADO). In a further development, the analytical solution of ADO was replaced by trajectory analysis, which resulted in capture theory. The calculations according to ADO and capture theory require precise knowledge of the dipole moment and the polarizability of the respective target molecule. However, for many relevant indoor related substances, these data are insufficiently known or not known at all. Consequently, the dipole moment *μ*_D_ and polarizability *α* of 114 organic compounds that are frequently found in indoor air had to be determined using advanced quantum mechanical methods. This required the development of an automated workflow that performs conformer analysis before computing *μ*_D_ and *α* using density functional theory (DFT). Then the reaction rate constants with the H_3_O^+^ ion are calculated according to the ADO theory (*k*_ADO_), capture theory (*k*_cap_) and advanced capture theory 
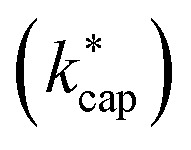
 for different conditions in the reaction chamber. The kinetic parameters are evaluated with regard to their plausibility and critically discussed for their applicability in PTR-MS measurements.

## Abstract 2

Proton transfer reaction rates were calculated for 114 organic compounds using ion–dipole collision theory with quantum mechanically determined dipole moments and polarizabilities.

## Graphical abstract

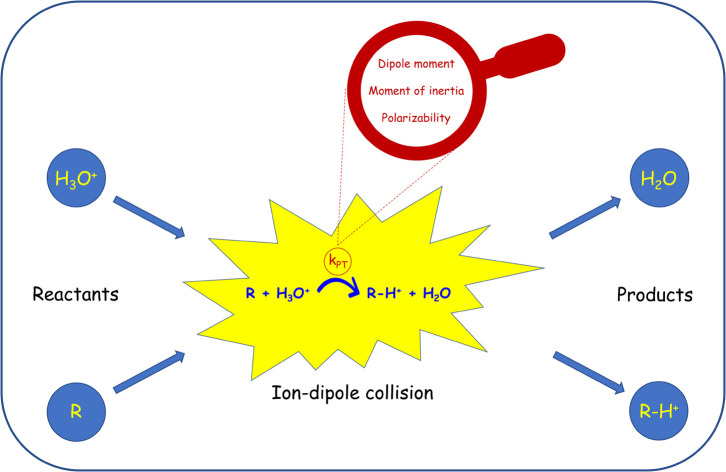

## 1 Introduction

A milestone in the analytical chemistry of the gas phase was the development of online mass spectrometry, which is, *inter alia*, suitable for numerous applications in food control, medicine and environmental research.^1,2^ The method was developed for very volatile and volatile organic compounds (VVOCs and VOCs) and is preferably used when their concentration has to be determined down to the ppt range and with high time resolution. The first applications in atmospheric chemistry aimed to examine ambient air.^3–5^ However, the suitability of online mass spectrometry for indoor applications was quickly recognized.^6,7^ A basic distinction is made between selected ion flow tube mass spectrometry (SIFT-MS)^8^ and proton-transfer-reaction mass spectrometry (PTR-MS),^9,10^ with the latter being mainly established in indoor air sciences.^7^ The use of PTR-MS to analyze human respiratory gas has been known for many years.^11–13^ Other successful areas of PTR-MS application and other mass spectrometry techniques with indoor-related questions involve ozone-initiated chemistry,^6,14–16^ photocatalytic reactions,^17,18^ measurements in museums,^19,20^ exposure under living conditions^21–23^ and gas phase/particle partitioning.^24^

However, online mass spectrometry has pros and cons. The coverage of a wide mass range and the high time resolution are often offset by calibration problems. In addition, only molecules whose proton affinity is greater than that of water can be studied, since H_3_O^+^ is the preferred reagent for proton transfer. Ions of the same nominal mass cannot be distinguished with a low-resolution quadrupole mass filter without disproportionate effort, their selective analysis requires a time-of-flight (ToF) detector. Not all molecules can be detected *via* the [M + 1]^+^ ion but rather fragment in the reaction tube, which makes both qualification and quantification more difficult.^25,26^

In a PTR-MS system quantification takes place from the signals of the ions involved, the device settings and the reaction constant of the proton transfer *k*_PT_. However, it is a fundamental problem of the PTR-MS method that reliable *k*_PT_ values are only available for a comparatively small number of molecules. On the other hand, *k*_PT_ can theoretically be calculated. The theory of ion–polar molecule collisions developed by Su and Bowers,^27,28^ also called average orientation dipole – ADO theory, is well suited for a reasonable estimation of reaction constants. In later works, Su and Chesnavich^29^ have taken an alternative route. The results of trajectory calculations were parameterized to give expressions which allow the calculation of *k*_PT_. This approach is known as capture theory. Finally, Su^30^ parameterized the trajectory calculations for the relative kinetic energy dependence of the rate constant at various temperatures. The ADO and the capture theory were discussed and compared by Ellis and Mayhew.^31^

Whichever theory is used, precise data on the dipole moment and polarizability of the target compound under the current reaction conditions are always required. The most critical quantity is the dipole moment. Even for a small and rigid molecule like acetaldehyde, the results of quantum mechanical calculations are between 2.65 D,^32^ 2.88 D^33^ and 2.94 D (this work), depending on the level of theory. Generally, the span between these quantum chemically calculated dipole moments increases with molecular size and flexibility, which can be attributed to routinely considering only a single molecular geometry. This ignores conformational flexibility and thermal effects, which is far from reality. These effects can significantly impact geometries and geometry-dependent properties like dipole moments (see below). For many molecules that play a role in the indoor environment, no reliable gas phase dipole moments are known at all.

In this work, we provide quantum chemically calculated, thermally averaged ensemble dipole moments and polarizabilities for 114 organic molecules. On that basis, we calculate and discuss rate constants for proton transfer reactions of organic compounds with H_3_O^+^ ions according to ADO theory and capture theory. Particular attention is paid to Su's^30^ advanced capture theory, because this allows the calculation of *k*_PT_ as a function of the electric field strength in the PTR-MS reaction chamber. We believe that these data are of particular value for the reliable determination of organic indoor air pollutants.

## 2 Methods

### 2.1 Software

The program OriginPro 2021b (64-bit) (OriginLab, Northampton, MA) was used for all non-quantum mechanical calculations, non-linear regression analyses, statistical evaluations and the drawing of some images. ChemDraw 16.0.1.4 (PerkinElmer Informatics Inc.) was used for drawing chemical structures. DFT calculations were performed using the program package TURBOMOLE 7.5.1.^34^

### 2.2 Quantum chemical calculations

Density functional theory (DFT) is routinely used to calculate dipole moments and polarizabilities for organic molecules. However, these calculations need reasonable starting geometries for all investigated compounds as input. Getting these geometries by hand for 114 organic compounds is tedious work and would limit further up-scaling of the developed workflow for an even larger number of compounds. Therefore, a workflow had to be developed as a first step that allows the automatic calculation of the relevant properties from more readily available inputs, such as CAS numbers.

**Fig. 1:**
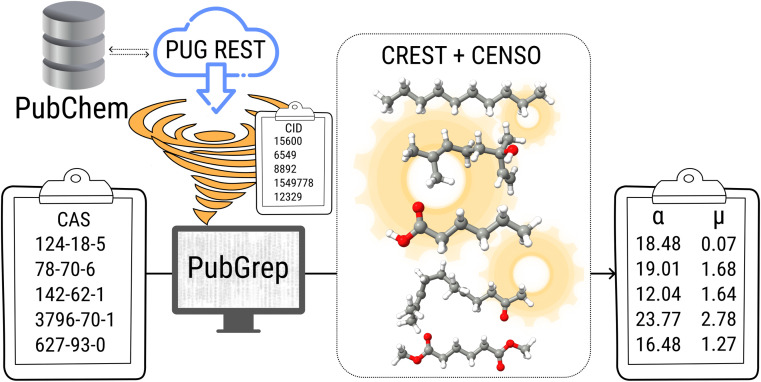
Simplified schematic of the automated calculation workflow including PubGrep, CREST and CENSO.

The PubChem^35^ database provides with its Power User Gateway (PUG) an URL-based API (application programming interface) for programmatic access to its contents. Using a simple command-line program dubbed PubGrep, we were able to get reasonable starting structures for all molecules by translating the given input to a PubChem Compound Identification (CID) first and afterward getting the corresponding 3D structure information from the database. If no 3D structure information was available, the 2D structure information was taken instead and converted into a 3D structure using the 2D to 3D structure converter included in our xTB program suite. Because dipole moments are highly dependent on the geometric structure of the molecule, a conformational sampling was performed using the program CREST^36^ with the GFN2-xTB^37^ semiempirical method. The resulting conformer ensemble was re-ranked with the program CENSO^38^ and the r^2^SCAN-3c^39^ composite DFT method with an energy threshold of 2.0 kcal mol^−1^ (8.4 kJ mol^−1^). These conformer ensembles were finally used to calculate the dipole moments and polarizabilities with the ωB97X-V^40^ DFT method. This functional is reported to perform very well for dipole moments with an RMS regularized error (RMSE) of just over 5% for small organic or inorganic molecules.^41^ A simplified schematic of this workflow is visualized in Fig. 1. Due to the rapid convergence of the computed dipole moment with the size of the applied AO basis (remaining completeness effects of about 0.1%), the def2-TZVPP basis set was chosen for computational efficiency. Instead of just using the lowest lying conformer for these calculations, the properties were calculated as a Boltzmann-weighted average over all conformers in the DFT refined ensemble at 298.15 K up to a Boltzmann threshold of 99% as1
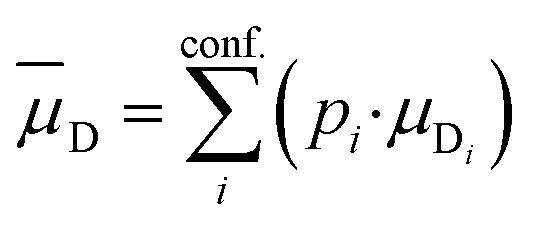
2
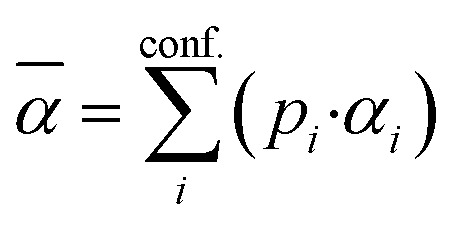
with the Boltzmann-weighting factor *p*_*i*_ as3
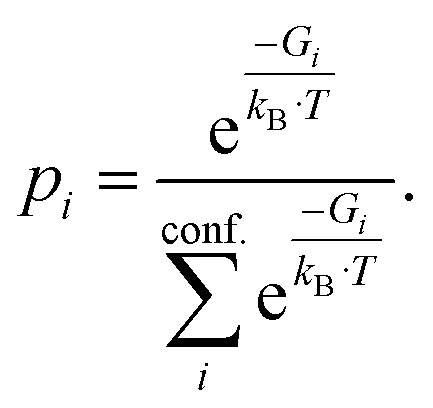
*G*_i_ is the DFT computed free energy of conformer *i, T* is the absolute temperature and *k*_*B*_ is the Boltzmann constant.

## 3 Fundamentals of PTR-MS

The principle of PTR-MS is based on chemical ionization of organic molecules. H_3_O^+^ ions are generated as primary reagents in an ion source. These enter the drift reaction chamber, where they collide with the sample gas molecules. In this process, a proton is transferred, resulting in the formation of ionized organic molecules and water as shown in eqn (4). Organic compounds, whose proton affinity is higher than the proton affinity of water (691 kJ mol^−1^)^42^ can be measured according to this principle.

4 R + H_3_O^+^ → RH^+^ + H_2_O

For [H_3_O^+^] >> [RH^+^] the VOC concentration [R] can be obtained from eqn (5) if the drift time *t*_r_ and the proton transfer constant *k*_PT_ are known.^9,10,31^5
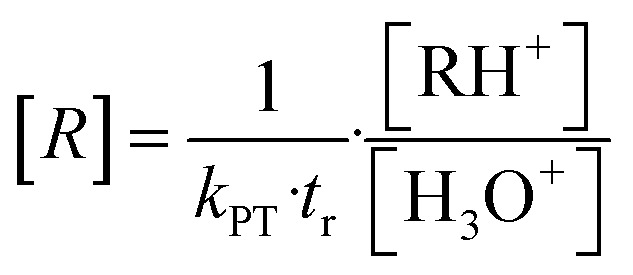
[RH^+^] and [H_3_O^+^] are the concentrations of the product and the primary ion, respectively. The drift time *t*_r_ and drift velocity *v*_d_ are related to the instrumental settings according to eqn (6).^33^6
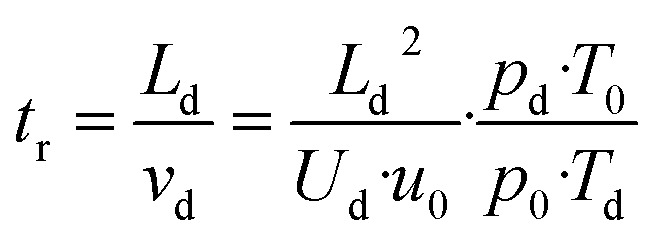
*L*_d_ is the length of the drift tube, *U*_d_ the applied drift voltage, and *u*_0_ is the reduced ion mobility,^5^ which itself depends on the actual instrument settings.^3,43^*T*_d_ and *p*_d_ are the actual temperature and pressure in the drift tube, *p*_0_ = 101 325 Pa and *T*_0_ = 273.15 K are the pressure and temperature at standard conditions, respectively. The conditions inside the drift tube are expressed by the ratio *E*/*N* of the electric field strength *E* = *U*_d_/*L*_d_ to the number density *N*. Assuming ideal gas behaviour we have *N* = *p*_d_/*k*_B_*T*_d_ and *N*_0_ = *p*_0_/*k*_B_*T*_0_, *k*_B_ is the Boltzmann constant. The drift velocity *v*_d_ from eqn (6) is given by eqn (7).7
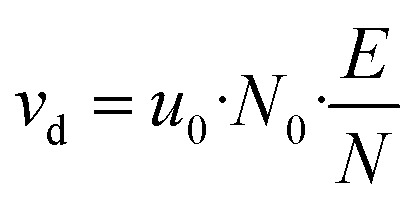


Usually, the ratio *E*/*N* is expressed in Townsend (Td) where 1 Td = 10^−21^ V m^2^. The values of *u*_0_ for H_3_O^+^ ions and the H_3_O^+^·(H_2_O) cluster were experimentally determined by Dotan *et al.*^43^ as a function of *E*/*N*. Note that these data can also be found as a graph in Warneke *et al.*^3^ and in the book of Ellis and Mayhew.^31^ In both publications, the data from Dotan *et al.*^43^ are discussed in detail and assessed as valid. Clusters only occur with small *E*/*N* values. For 100 Td the H_3_O^+^·(H_2_O)/H_3_O^+^ concentration ratio is 1.1 and for 120 Td it is 0.023.^31^Table 1 shows values for *u*_0_, *v*_d_ and *t*_r_ at different *E*/*N* with a drift tube length *L*_d_ = 9.6 cm. Clusters are not discussed further, the data are given in Table 1 for comparison. Small molecules have similar rate constants for H_3_O^+^ and H_3_O^+^·(H_2_O).^44^ The *E*/*N* value not only affects the energy of the ions in the drift chamber, but also the fragmentation behavior of the molecules. In chemical ionization, the [M + 1]^+^ ions are often formed preferentially, but many molecules tend to fragment, a well-studied example being terpenoids.^25,45^ Normally, molecules fragment more with higher *E*/*N*. A comprehensive fragment ion database is provided by Pagonis *et al.*^46^

**Table 1:** Data for H_3_O^+^ and H_3_O^+^·(H_2_O) in the PTR-MS drift tube at *L*_d_ = 9.6 cm and *T*_d_ = 353 K. The *u*_0_ were taken from Warneke *et al.*^3^

Ion	*E*/*N* T_d_	*u*_0_ cm^2^ (V s)^−1^	*v*_d_ m s^−1^	*t*_r_ μs	KE_ion_ 10^−20^ J	KE_ion_ eV
H_3_O^+^	80	2.73	587	164	2.10	0.13
H_3_O^+^	90	2.74	663	145	2.47	0.15
H_3_O^+^	100	2.75	739	130	2.90	0.18
H_3_O^+^	110	2.80	828	116	3.45	0.22
H_3_O^+^	120	2.81	906	106	3.99	0.25
H_3_O^+^	130	2.85	996	96	4.67	0.29
H_3_O^+^	140	2.89	1087	88	5.42	0.34
H_3_O^+^·H_2_O	80	2.32	499	193	1.72	0.11
H_3_O^+^·H_2_O	90	2.36	571	168	2.02	0.13
H_3_O^+^·H_2_O	100	2.41	648	148	2.40	0.15
H_3_O^+^·H_2_O	110	2.44	721	133	2.80	0.17

## 4 Determination of *k*_PT_ values

### 4.1 Calculation of the reaction rate constant *k*_ADO_ from dipole orientation theory

A widely used approach to calculate *k*_PT_ follows the ion–dipole collision theory developed by Su and Bowers.^27^Eqn (8) is an extension of Langevin's approach. The term *k*_L_ describes the classical Langevin rate coefficient for the interaction of an ion with a non-polar molecule. The contribution of the interaction between ion and dipole is given by *k*_*μ*_D__.8
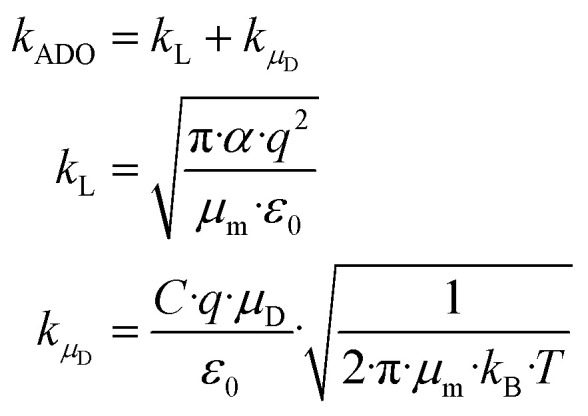
In eqn (8), *q* is the charge of the ion, *α* the polarizability of the neutral reactant with dipole moment *μ*_D_, *μ*_m_ the reduced mass of the reactants and *ε*_0_ the vacuum electric permittivity. *C* is a temperature dependent dimensionless “locking” parameter (0 ≤ *C* ≤ 1) that accounts for the permanent dipole moment of the molecule. All quantities are given in SI units. Conversion factors to other units as well as values of the fundamental constants are given in the ESI.†

### 4.2 The locking parameter *C*

**Fig. 2:**
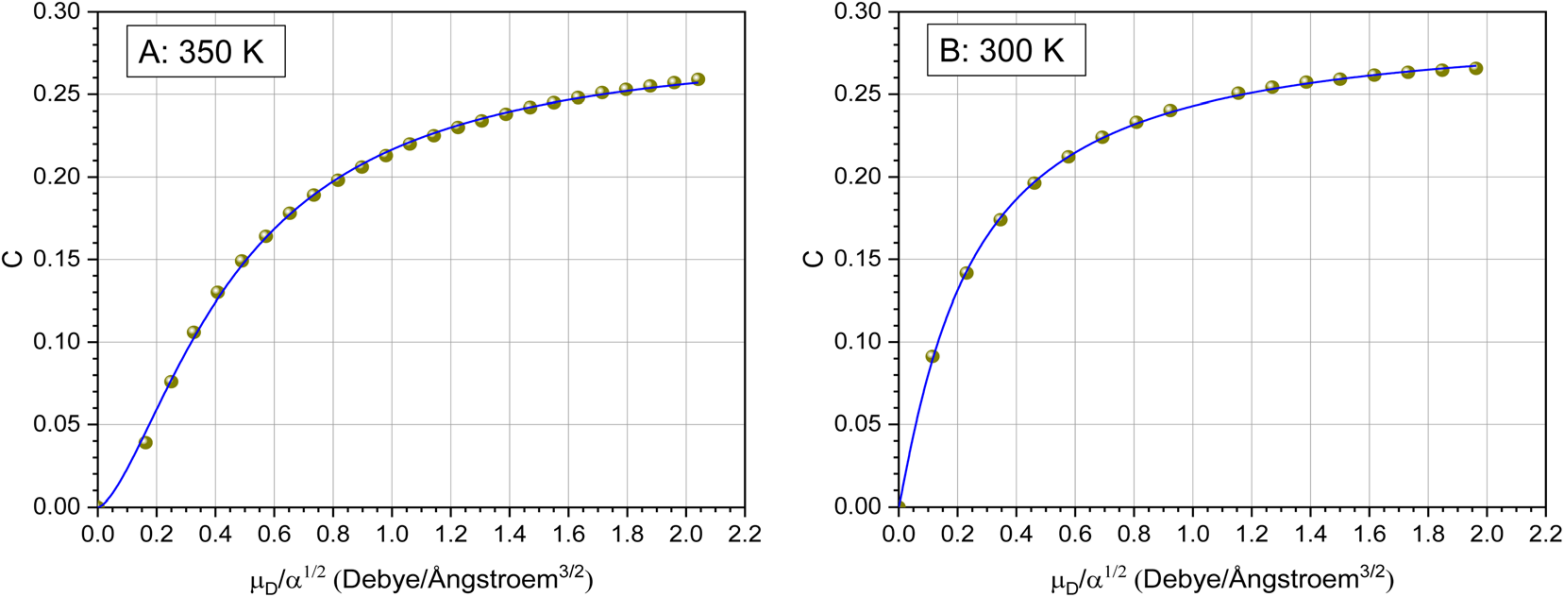
Plot of the locking parameter *C versus μ*_D_/*α*^1/2^ and fitting curve with eqn (9). The data for 350 K (A) are from Su and Bowers^47^ and the 300 K data (B) are from Su *et al.*^48^

*C* is a function of the dipole moment *μ*_D_ and the square root of the polarizability *α* of the neutral molecule. For an entirely non-polar molecule, *C* = 0 and then eqn (8) corresponds to the classical Langevin approach. Temperature-dependent values for *C* as a function of *μ*_D_/*α*^1/2^ were published by Su and Bowers^47^ and Su *et al.*^48^ The data sets for 300 K and 350 K were fitted with the empirical eqn (9) in order to be able to calculate *k*_ADO_ for the respective target molecules. The fit parameters are presented in Table 2, the fit curves are shown in Fig. 2 and the data are provided in the ESI (Appendix A).†9
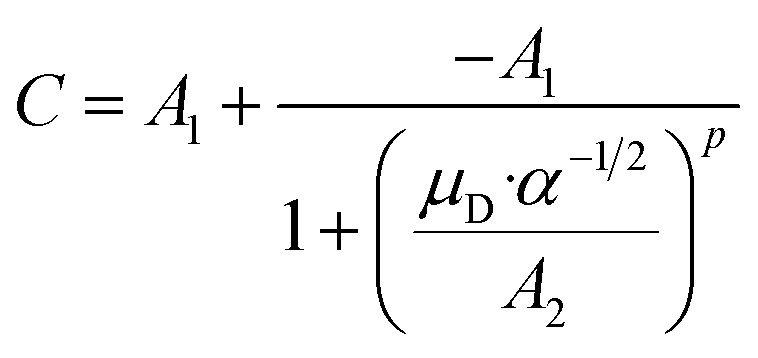


**Table 2:** Fit parameters according to eqn (9) for the data sets *C* = *f*(*μ*_D_, *α*) (see ESI) at 300 K and 350 K. Here *μ*_D_ is given in Debye and *α* in 10^−24^ cm^3^

Temperature	*A*_1_	*A*_2_	*p*	Data from ref.
300 K	0.29366	0.24274	1.10725	Su *et al.*^48^
350 K	0.28279	0.46805	1.56293	Su and Bowers^47^

Extending their ADO theory, Su *et al.*^48^ developed an approach that takes into account the moment of inertia of a molecule. The authors explain that, strictly speaking, their extended theory only provides exact values for small linear molecules with a moment of inertia I ≤ 10^−39^ g cm^2^. Moreover, Su *et al.*^48^ mention that the temperature dependent *C* values from Su and Bowers^47^ contain a “minor error”. Unfortunately, corrected data for 350 K were not presented, only for 300 K (see Fig. 2B). We therefore used the data from Su and Bowers^47^ in our work in order to be able to make comparisons with other *k*_PT_ values.

### 4.3 Calculation of the collision rate constant *k*_cap_ by trajectory analysis

An alternative to analytical expressions for calculating rate coefficients for ion–molecule interactions is to model reaction processes by trajectory calculations. Su and Chesnavich^29^ have published corresponding parameterizations of this method, known as capture theory, with which temperature-dependent rate constants can be determined. First, the reaction rate *k*_L_ according to Langevin (see eqn (8) with *C* = 0) is determined. In order to obtain the capture rate coefficient *k*_cap_, *k*_L_ is multiplied by the parameterized quantity *K*_cap_ according to eqn (10) and (11).

10 *k*_cap_ = *k*_L_·*K*_cap_(*T*_R_)

11
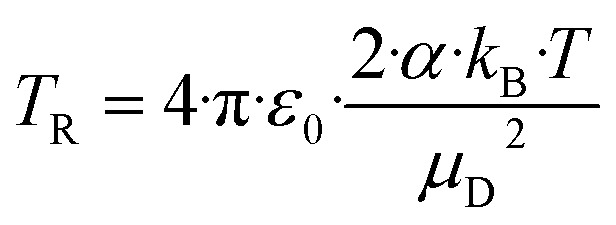


All quantities are given in SI units. The full trajectory analysis method for *K*_cap_ is provided with the respective units in the ESI (Appendix B).† It is a disadvantage of Su and Chesnavich's^29^ capture theory that only for small molecules *K*_cap_ is a function of *T*_R_ and independent of the moment of inertia *I*. Ellis and Mayhew^31^ state that for the reactions taking place in the PTR-MS, *K*_cap_ is insensitive to the moment of inertia. In principle, however, this must be checked individually for each molecule. The question whether the conditions of the capture theory are fulfilled depends not only on *I* but also on *μ*_D_ and *α*. We have not performed a rigorous analysis of all the compounds being relevant here, as *k*_cap_ is presented and calculated for comparison purposes only.

### Calculation of the collision rate constant 
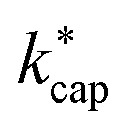
 by advanced trajectory analysis

4.4

In a later work, Su^30^ presented improved parameterizations that cover a wider temperature range. This takes into account the effect that the temperature of the ions traveling along a drift tube is higher than the drift tube temperature *T*_d_ because they experience additional energy from the electric field. Eqn (12) for the total mean ion kinetic energy KE_ion_ was originally developed by Wannier^49^ and later discussed by de Gouw *et al.*^5^ in relation to PTR-MS applications.12
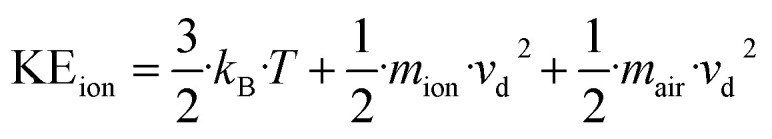


For *T* = 353 K and different *E*/*N* ratios, KE_ion_ values of H_3_O^+^ and H_3_O^+^·H_2_O are listed in Table 1. The collision energy of an ion–molecule reaction is then obtained from eqn (13). This is the kinetic energy KE_CM_ relative to the center of mass of the colliding system that is available for the reaction process.^31^13
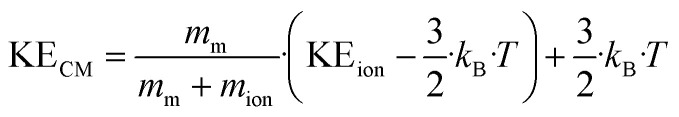


In eqn (12) and (13)*m*_ion_ is the mass of the respective ion H_3_O^+^ or H_3_O^+^·H_2_O, *m*_air_ is the average mass of dry air and *m*_m_ is the mass of the target molecule. The reaction constant 
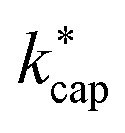
 is then obtained according to eqn (14) from the Langevin constant *k*_L_ and the factor *K*_C_, which according to Su^30^ depends on the parameters *τ* and *ε* to be calculated from the dipole moment *μ*_D_, polarizability *α* and KE_CM_.14
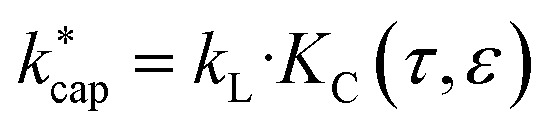


The full trajectory analysis method is provided in the ESI (Appendix C).† The influence of the kinetic energy on the reaction constant is discussed by Ellis and Mayhew^31^ using acetone at 300 K as an example. The difference to thermal energy is also significant for the increased temperature in a PTR-MS drift tube (353 K was assumed here). A value of KE_CM_ = 0.20 eV then results for acetone at *E*/*N* = 120 Td, the thermal energy from 3/2·*k*_B_·*T* accounts to 0.05 eV. As already pointed out in detail by Cappellin *et al.*,^50^ the particular advantage of 
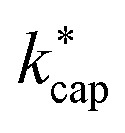
 is that it can be used over a wide temperature range. Moreover, there is a direct connection with the electric field in the drift tube *via* the drift velocity *v*_d_, see eqn (7). This allows the rate constants to be calculated as a function of the *E*/*N* value for the respective drift tube temperature.

## 5 Results and discussion

### 5.1 Dipole moments

The essential parameters for calculating *k*_ADO_, *k*_cap_ and 
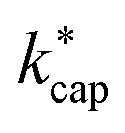
 are the dipole moment *μ*_D_ and the polarizability *α*. Both quantities are linked to the molar polarization *via* the Debye equation.^51^ For PTR-MS measurements, values are required for the complete, thermally averaged conformer ensemble in the gas phase, because changes in the molecular geometry affect the dipole moment in particular. This effect has not been considered so far in our context. It has already been mentioned that published values can vary considerably. For gas phase 2-butoxyethanol, we calculate a dipole moment of 2.42 D after conformer analysis, while Iglesias *et al.*^52^ published 2.13 D for this molecule in cyclohexane. For 2-ethyl-1-hexanol with a calculated gas phase dipole moment of 1.61 D and a measured dipole moment in cyclohexane of 1.69 D at 298 K^53^ the difference is less pronounced. Nevertheless, it should be noted that the geometry of a molecule significantly impacts the dipole moment, and solvent interactions can influence the conformational distribution.^38^ Therefore, values measured in solution are not useful for flexible molecules. For example, the absolute difference between the dipole moment for the thermally averaged conformer ensemble and the lowest-lying conformer (Δ*μ*_D_) of 2-ethyl-1-hexanol is 0.21 D. The mean deviation of the Δ*μ*_D_ values for all of the investigated compounds is with 0.13 D significant. The deviations are more pronounced for non-rigid molecules, such as 2-butoxyethylacetate (0.43 D), geranylacetone (0.45 D) or dimethylsebacate (1.4 D). The maximum Δ*μ*_D_ value is as high as 2.62 D for dimethyl phthalate. Moreover, tabulated data often has the disadvantage that the experimental conditions are unknown. Reliable data are only tabulated for a limited number of molecules,^54,55^ but quantum chemical tools for their calculation are nowadays routinely available.^50^ In this respect, quantum mechanical calculations computed for conformer ensembles appear as the only reasonable alternative, especially in the case of large and non-rigid molecules. The polarizabilities are much less susceptible to conformer changes than the dipole moments. Since the quality of many of the dipole moments available in the literature could not be evaluated and in order to avoid inconsistencies, only dipole moments and polarizabilities that were determined quantum mechanically according to the method described in Section 2 were used to calculate *k*_ADO_, *k*_cap_ and 
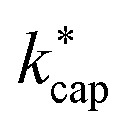
. All dipole moments, polarizabilities and calculated 
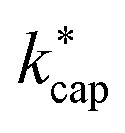
 values are listed in Table 3. The *k*_ADO_ and *k*_cap_ values are listed in the ESI† (Appendix D).

**Table 3:** Dipole moments, polarizabilities and calculated rate constants 
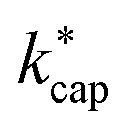
 at 353 K in dependence of *E*/*N* for the reaction of organic compounds with H_3_O^+^ ions in the PTR-MS drift tube according to Su's^30^ capture theory. Other parameters and rate constants are listed in the ESI

Compound	CAS	*μ*_D_ D	*α* 10^−24^ cm^3^	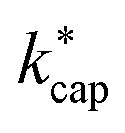 10^−9^ cm^3^ s^−1^						
				(*E*/*N*) = 80 Td	90	100	110	120	130	140
**Aliphatic and cyclic hydrocarbons**										
*n*-Hexane	110-54-3	0.04	11.30	1.99	1.99	1.99	1.99	1.99	1.99	1.99
*n*-Heptane	142-82-5	0.08	13.09	2.12	2.12	2.12	2.12	2.12	2.12	2.12
*n*-Octane	111-65-9	0.06	14.87	2.24	2.24	2.24	2.24	2.24	2.24	2.24
*n*-Nonane	111-84-2	0.08	16.66	2.35	2.35	2.35	2.35	2.35	2.35	2.35
*n*-Decane	124-18-5	0.07	18.48	2.46	2.46	2.46	2.46	2.46	2.46	2.46
Cyclohexane	110-82-7	0.00	10.36	1.91	1.91	1.91	1.91	1.91	1.91	1.91
Methylcyclohexane	108-87-2	0.12	12.17	2.05	2.05	2.05	2.05	2.05	2.05	2.05
4-Vinyl cyclohexene (4-VCH)	100-40-3	0.30	13.82	2.17	2.17	2.17	2.17	2.17	2.17	2.17

**Aromatic hydrocarbons**										
Benzene	71-43-2	0.00	10.07	1.90	1.90	1.90	1.90	1.90	1.90	1.90
Toluene	108-88-3	0.39	12.00	2.04	2.04	2.04	2.04	2.04	2.04	2.04
Ethylbenzene	100-41-4	0.42	13.80	2.17	2.17	2.17	2.17	2.17	2.17	2.17
*o*-Xylene	95-47-6	0.66	13.85	2.24	2.17	2.17	2.17	2.17	2.17	2.17
*m*-Xylene	108-38-3	0.37	13.95	2.18	2.18	2.18	2.18	2.18	2.18	2.18
*p*-Xylene	106-42-3	0.00	14.00	2.18	2.18	2.18	2.18	2.18	2.18	2.18
1,2,3-Trimethylbenzene	526-73-8	0.73	15.70	2.36	2.29	2.29	2.29	2.29	2.29	2.29
1,2,4-Trimethylbenzene	95-63-6	0.40	15.85	2.30	2.30	2.30	2.30	2.30	2.30	2.30
1,3,5-Trimethylbenzene	108-67-8	0.00	15.91	2.31	2.31	2.31	2.31	2.31	2.31	2.31
Isopropylbenzene	98-82-8	0.39	15.56	2.28	2.28	2.28	2.28	2.28	2.28	2.28
Styrene	100-42-5	0.17	14.43	2.22	2.22	2.22	2.22	2.22	2.22	2.22
Chlorobenzene	108-90-7	1.86	12.25	2.86	2.75	2.65	2.56	2.49	2.42	2.37
1,2-Dichlorobenzene	95-50-1	2.65	14.32	3.51	3.43	3.33	3.20	3.08	2.94	2.84
1,4-Dichlorobenzene	106-46-7	0.00	14.56	2.18	2.18	2.18	2.18	2.18	2.18	2.18
4-Phenyl cyclohexene (4-PCH)	4994-16-5	0.28	20.06	2.55	2.55	2.55	2.55	2.55	2.55	2.55

**Polycyclic aromatic hydrocarbons**										
Naphthalene	91-20-3	0.00	17.37	2.40	2.40	2.40	2.40	2.40	2.40	2.40
1-Methylnaphthalene	90-12-0	0.36	19.25	2.51	2.51	2.51	2.51	2.51	2.51	2.51
1-Chloronaphthalene	90-13-1	1.79	19.50	3.03	2.96	2.89	2.83	2.79	2.75	2.72
Anthacene	120-12-7	0.00	26.19	2.89	2.89	2.89	2.89	2.89	2.89	2.89
Phenanthrene	85-01-8	0.01	24.89	2.82	2.82	2.82	2.82	2.82	2.82	2.82

**Terpenoids**										
Isoprene	78-79-5	0.27	9.88	1.91	1.91	1.91	1.91	1.91	1.91	1.91
α-Pinene	80-56-8	0.18	16.51	2.33	2.33	2.33	2.33	2.33	2.33	2.33
β-Pinene	127-91-3	0.72	16.70	2.41	2.34	2.34	2.34	2.34	2.34	2.34
3-Carene	13466-78-9	0.17	16.74	2.35	2.35	2.35	2.35	2.35	2.35	2.35
d-Limonene	5989-27-5	0.57	17.45	2.39	2.39	2.39	2.39	2.39	2.39	2.39
α-Phellandrene	4221-98-1	0.24	17.42	2.39	2.39	2.39	2.39	2.39	2.39	2.39
Myrcene	123-35-3	0.44	18.73	2.48	2.48	2.48	2.48	2.48	2.48	2.48
β-Caryophyllene	87-44-5	0.50	25.07	2.81	2.81	2.81	2.81	2.81	2.81	2.81
Terpinolene	586-62-9	0.24	17.81	2.42	2.42	2.42	2.42	2.42	2.42	2.42
Linalool	78-70-6	1.68	19.01	2.95	2.88	2.82	2.77	2.73	2.69	2.67
α-Terpineol	98-55-5	1.52	18.02	2.79	2.74	2.69	2.65	2.62	2.59	2.57

**Alcohols**										
Methanol	67-56-1	1.70	3.14	2.37	2.33	2.30	2.26	2.23	2.18	2.13
Ethanol	64-17-5	1.73	4.90	2.47	2.43	2.39	2.33	2.27	2.19	2.11
1-Propanol	71-23-8	1.68	6.64	2.51	2.46	2.40	2.31	2.23	2.14	2.06
2-Propanol	67-63-0	1.69	6.66	2.52	2.47	2.41	2.32	2.24	2.15	2.07
1-Butanol	71-36-3	1.68	8.39	2.59	2.52	2.43	2.33	2.25	2.18	2.12
2-Butanol	75-65-0	1.69	8.37	2.59	2.53	2.44	2.34	2.26	2.19	2.13
2-Methyl-1-propanol	78-83-1	1.61	8.37	2.53	2.46	2.37	2.27	2.20	2.14	2.08
1-Pentanol	71-41-0	1.65	10.17	2.64	2.53	2.44	2.36	2.30	2.24	2.19
1-Hexanol	111-27-3	1.66	11.96	2.69	2.59	2.51	2.44	2.38	2.33	2.29
1-Heptanol	111-70-6	1.66	13.75	2.74	2.65	2.58	2.51	2.47	2.42	2.38
2-Ethyl-1-hexanol	104-76-7	1.61	15.37	2.75	2.68	2.62	2.56	2.52	2.48	2.45
Benzyl alcohol	100-51-6	1.62	12.68	2.67	2.58	2.51	2.44	2.40	2.35	2.31

**Aldehydes**										
Acetaldehyde	75-07-0	2.94	4.45	3.40	3.33	3.26	3.19	3.13	3.07	3.02
Propanal	123-38-6	2.85	6.14	3.35	3.29	3.23	3.17	3.12	3.05	2.99
Butanal	123-72-8	2.83	7.90	3.39	3.34	3.29	3.22	3.16	3.08	2.99
2-Methylpropanal	78-84-2	2.91	7.88	3.45	3.39	3.34	3.27	3.21	3.14	3.05
Pentanal	110-62-3	2.85	9.68	3.49	3.43	3.37	3.30	3.22	3.12	3.00
Hexanal	66-25-1	2.83	11.45	3.55	3.49	3.43	3.33	3.24	3.12	2.98
Heptanal	111-71-7	2.86	13.25	3.66	3.59	3.51	3.40	3.29	3.15	3.01
Octanal	124-13-0	2.85	15.04	3.72	3.65	3.55	3.43	3.30	3.15	3.03
Nonanal	124-19-6	2.85	16.84	3.79	3.71	3.60	3.46	3.32	3.18	3.07
Decanal	112-31-2	2.85	18.66	3.86	3.76	3.64	3.49	3.35	3.22	3.12
Acrolein (trans)	107-02-8	3.43	6.34	3.84	3.76	3.68	3.60	3.54	3.47	3.41
Acrolein (cis)	107-02-8	2.81	6.14	3.33	3.27	3.22	3.16	3.11	3.04	2.97
*trans*-2-Butenal	123-73-9	4.20	8.40	4.48	4.38	4.29	4.19	4.11	4.03	3.95
*trans*-2-Hexenal	6728-26-3	4.37	12.02	4.68	4.59	4.50	4.41	4.33	4.24	4.15
Furfural (trans)	98-01-1	3.60	9.82	3.99	3.91	3.85	3.77	3.70	3.62	3.53
Furfural (cis)	98-01-1	4.31	9.88	4.51	4.41	4.32	4.22	4.15	4.07	3.98
Glyoxal (cis)	107-22-2	3.68	4.64	3.87	3.77	3.68	3.58	3.51	3.43	3.36
Benzaldehyde	100-52-7	3.42	12.53	4.01	3.95	3.88	3.79	3.71	3.60	3.47

**Ketones**										
Acetone	67-64-1	3.11	6.15	3.55	3.48	3.42	3.35	3.29	3.23	3.16
2-Butanone (MEK)	78-93-3	2.97	7.86	3.49	3.44	3.38	3.31	3.25	3.18	3.09
4-Methyl-2-pentanone (MIBK)	108-10-1	2.87	11.36	3.58	3.52	3.45	3.36	3.27	3.15	3.01
Cyclohexanone	108-94-1	3.45	10.57	3.93	3.86	3.79	3.72	3.65	3.56	3.45
6-Methyl-5-heptene-2-one (6-MHO)	110-93-0	2.88	15.12	3.75	3.68	3.58	3.46	3.34	3.18	3.05
4-Oxopentanal (4-OPA)	626-96-0	2.95	9.73	3.51	3.45	3.39	3.32	3.24	3.14	3.03
Geranylacetone	3796-70-1	2.78	23.77	3.98	3.85	3.69	3.54	3.44	3.34	3.25
3-Octanone	106-68-3	2.71	14.90	3.62	3.53	3.44	3.31	3.18	3.04	2.93

**Esters**										
Methyl acetate	79-20-9	1.88	6.77	2.61	2.56	2.50	2.42	2.34	2.23	2.14
Ethyl acetate	141-78-6	2.03	8.59	2.82	2.76	2.69	2.59	2.49	2.39	2.30
*n*-Butyl acetate	123-86-4	2.06	12.13	3.00	2.92	2.81	2.69	2.60	2.52	2.45
2-Ethylhexyl acetate	103-09-3	1.84	19.01	3.04	2.95	2.88	2.82	2.77	2.73	2.69
Dimethyl phthalate	131-11-3	2.93	19.46	3.91	3.81	3.68	3.52	3.39	3.26	3.15
Diethylphthalate	84-66-2	2.60	23.06	3.79	3.63	3.50	3.37	3.28	3.19	3.12
Dimethyl succinate	106-65-0	1.30	12.80	2.38	2.33	2.29	2.25	2.22	2.20	2.17
Dimethyl adipate	627-93-0	1.27	16.48	2.55	2.52	2.48	2.46	2.43	2.41	2.40
Dimethyl sebacate	106-79-6	1.50	23.76	3.01	2.97	2.93	2.90	2.88	2.86	2.84

**Glycols**										
2-Ethoxyethanol	110-80-5	2.45	9.20	3.16	3.10	3.04	2.95	2.87	2.76	2.63
1-Methoxy-2-propanol	107-98-2	2.33	9.16	3.07	3.01	2.95	2.86	2.77	2.65	2.53
1,2-Propanediol	57-55-6	2.34	7.29	2.97	2.93	2.87	2.80	2.74	2.64	2.54
2-Butoxyethanol	111-76-2	2.42	12.70	3.30	3.22	3.13	3.01	2.89	2.77	2.68
2-Butoxyethoxyethanol	54446-78-5	2.81	16.93	3.74	3.65	3.54	3.39	3.26	3.13	3.02
Phenoxyethanol	122-99-6	1.67	15.41	2.79	2.71	2.64	2.58	2.54	2.50	2.46
2-Butoxyethyl acetate	112-07-2	1.73	16.36	2.85	2.77	2.70	2.64	2.59	2.55	2.52

**Acids**										
Formic acid	64-18-6	1.56	3.30	2.13	2.10	2.07	2.02	1.98	1.92	1.86
Acetic acid	64-19-7	1.80	5.01	2.44	2.40	2.35	2.29	2.23	2.15	2.06
Propionic acid	79-09-4	1.69	6.72	2.46	2.41	2.34	2.25	2.17	2.08	2.01
Hexanoic acid	142-62-1	1.64	12.04	2.64	2.55	2.47	2.40	2.35	2.30	2.26
Isobutyric acid	79-31-2	1.74	8.45	2.59	2.52	2.44	2.33	2.25	2.18	2.11

**Phenones**										
Acetophenone	98-86-2	3.16	14.20	3.90	3.84	3.76	3.65	3.55	3.41	3.25
Benzophenone	119-61-9	3.11	22.62	4.19	4.08	3.95	3.77	3.63	3.49	3.38
Darocur 1173	7473-98-5	3.89	18.41	4.55	4.47	4.38	4.27	4.16	4.01	3.84
Irgacure 184	947-19-3	3.42	22.90	4.40	4.30	4.17	4.01	3.85	3.68	3.54

**Siloxanes**										
D4	556-67-2	0.60	28.26	2.94	2.94	2.94	2.94	2.94	2.94	2.94
D5	541-02-6	0.86	35.16	3.26	3.26	3.26	3.26	3.26	3.26	3.26
D6	540-97-6	0.74	42.11	3.56	3.56	3.56	3.56	3.56	3.56	3.56

**Other**										
Acetonitrile	75-05-8	4.06	4.34	4.39	4.28	4.17	4.05	3.96	3.86	3.77
Ethylamine	75-04-7	1.37	5.55	2.27	2.21	2.15	2.07	2.00	1.93	1.87
Diethylamine	109-89-7	0.99	9.17	2.07	2.04	2.01	1.98	1.96	1.94	1.93
Triethylamine	121-44-8	0.68	12.56	2.15	2.14	2.07	2.07	2.07	2.07	2.07
Dimethyl sulfide	75-18-3	1.68	7.27	2.56	2.50	2.43	2.33	2.25	2.17	2.10
1,3-Benzothiazol	95-16-9	1.31	15.49	2.56	2.51	2.48	2.44	2.42	2.40	2.38
*N*-Methyl-2-pyrrolidon	872-50-4	4.12	10.28	4.38	4.29	4.21	4.12	4.05	3.97	3.88
2-Butanonoxim	96-29-7	1.04	9.54	2.09	2.05	2.02	1.99	1.97	1.95	1.94
Phenol	108-95-2	1.29	10.87	2.33	2.28	2.23	2.19	2.15	2.12	2.10

However, if one refers to the literature, the question now arises as to how precisely the dipole moment of a molecule is available for the conditions prevailing here and what range has to be expected when calculating the reaction constants. For example, experimental values of 1.84 D^55^ and 1.87 D^56^ in the liquid state have been published for *n*-butyl acetate. Our calculations resulted in a value of *μ*_D_ = 2.06 D. The difference between experiment (liquid phase) and theory (gas phase) is striking but not surprising. Dipole moments in gas phase and liquid phase are hardly comparable. Molecular geometries are impacted by dispersion and solvent interactions and will therefore often look very different in these phases, changing the resulting dipole moments.

Attig *et al.*^57^ calculated dipole moments of *n*-butyl acetate using quantum mechanical methods with 18 different levels of theory. The minima and maxima were 1.97 D and 2.35 D, respectively. However, these values are obtained only for the lowest-lying conformer and are not directly comparable to our thermally averaged results over a conformer ensemble. The computed dipole moment for the lowest-lying conformer in our ensemble is 2.15 D, *i.e.*, very close to the calculated value of Attig *et al.*^57^ (2.18 D) using the B3LYP/6-311++G(d,p) level of theory, which is arguably the best of their methods. A more recent benchmark study on the accuracy of dipole moments for DFT calculations^41^ suggests a root mean square error (RMSE) of about 5% for the here employed method (see Section 2.1) which is quite close to the assumption by Cappellin *et al.*^50^ regarding the uncertainty in the quantum chemical calculation for the dipole moment.

**Fig. 3:**
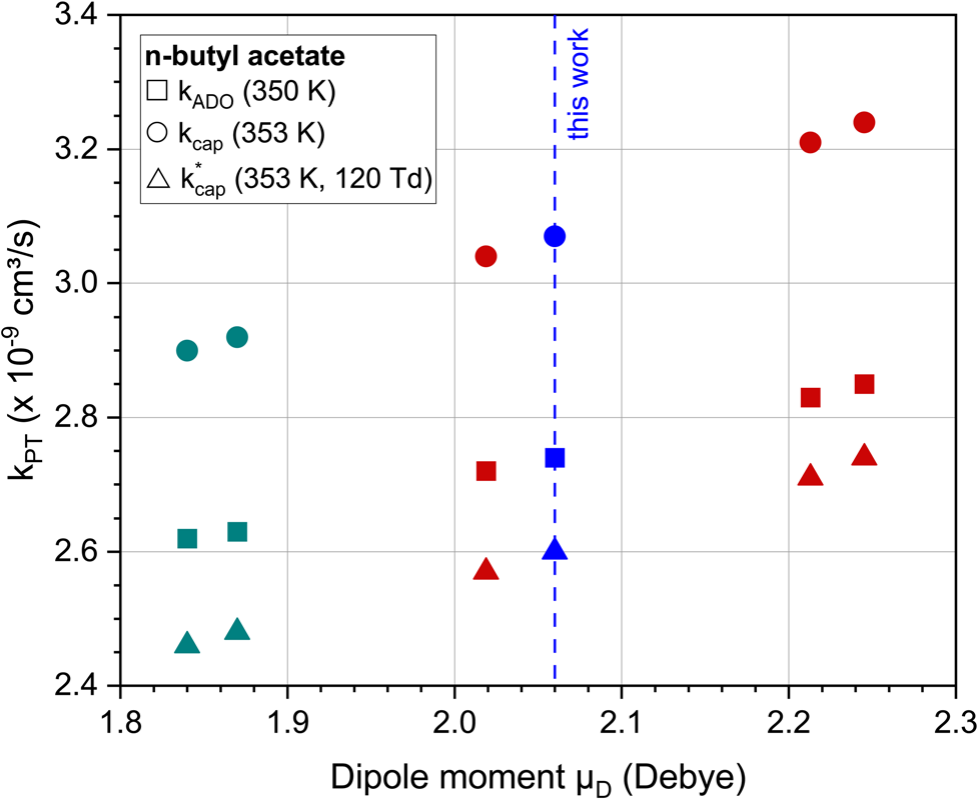
Rate constants *k*_ADO_, *k*_cap_ and 
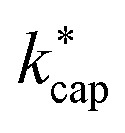
 for the reaction of *n*-butyl acetate with H_3_O^+^ ions as a function of the dipole moment. For all calculations the polarizability was *α* = 12.13·10^−24^ cm^3^. The values of 1.84 D^55^ and 1.87 D^56^ (green) are in cyclohexane (note: these data are used for purposes of comparison only); 2.06 D (blue) is this work; 2.02 D, 2.21 D and 2.25 D (red) are calculated data from Attig *et al.*^57^ for three assigned conformers.

Fig. 3 shows the dependence of the *k*_PT_ values, which were calculated with the three methods presented, on the dipole moment using the example of *n*-butyl acetate. For comparison with our value of 2.06 D, the experimentally determined dipole moments in cyclohexane^55,56^ and calculated dipole moments by Attig *et al.*^57^ for three assigned conformers were taken. Regardless of the calculation method, the rate constants for this molecule vary by 5–10% when the dipole moments differ by about 10%. However, it should be noted that uncertainties in the polarizability were not considered. For *n*-butyl acetate, Fig. 3 shows reasonable values for the dependence of *k*_PT_ on the dipole moment. However, the results must not be generalized, since higher dipole moments have a greater influence on *k*_PT_. The differences between the calculation methods are discussed in Section 5.2.

**Fig. 4:**
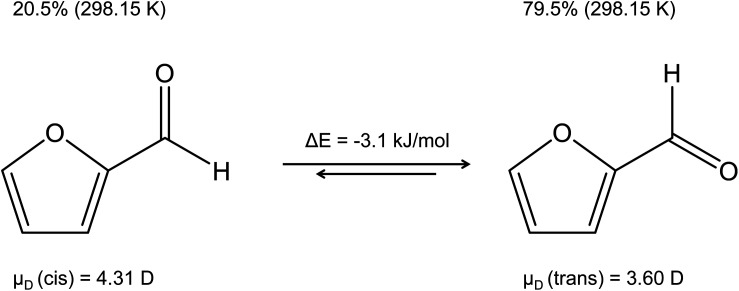
Dipole moments of the cis and trans isomers of furfural in the gas phase,^58^ percent of each isomer, and the energy difference between the two conformers.^59^

The reliability of theoretical *k*_PT_ calculations stands and falls not only with the precision of the dipole moment and the polarizability, but it is also important to get the “right” molecule. The question may therefore arise as to which isomer a dipole moment is to be calculated for. A good example is furfural shown in Fig. 4, whose dominant trans isomer at 298.15 K has a calculated dipole moment of 3.60 D, while 4.31 D is given for the cis isomer.^58^ Moreover, the energy of the trans isomer is 3.1 kJ mol^−1^ lower than that of the cis isomer.^59^ A similar case concerns acrolein with the more abundant cis isomer^60^ (*μ*_D_(cis) = 2.81 D). The dipole moment of the trans isomer is *μ*_D_(trans) = 3.43 D. Acrolein was already discussed in a previous publication.^61^

### 5.2 Reaction rate constants

Application of the ion–dipole collision theory is the most common method for determining *k*_PT_ in PTR-MS and SIFT measurements. It should only be mentioned at this point that other formulas can be used to calculate reaction constants in related techniques such as multiple-ion laminar flow tube spectrometry (MIFT).^62,63^

Zhao and Zhang^32^ provide *k*_ADO_ values at 300 K for a total of 136 substances. However, Cappellin *et al.*^33^ doubt that these data can be used for PTR-MS applications and argue with the electric field strength, which induces far more energetic collisions than those at room temperature. Blake *et al.*^10^ point out that the ADO theory tends to result in lower rate constants than experimentally determined *k*_PT_ values. With reference to Wannier^49^ it is assumed that the effective temperature for ion–molecule collisions is higher than the temperature in the drift tube. For an ion with a drift velocity in the range of 900 m s^−1^, the thermal energy accounts for only a small part of the total kinetic energy. The calculation of the *k*_ADO_ data listed in the ESI† was carried out according to Su and Bowers^27,28^ using their data for the locking parameter *C* at 350 K,^47^ a common temperature for PTR-MS measurements. It was already mentioned that Su *et al.* critically discussed these data in a later publication.^48^

Ellis and Mayhew^31^ also state that the ADO theory underestimates experimental *k*_PT_ values at 300 K, but found good agreement with *k*_cap_ values calculated according to Su and Chesnavich.^29^ This is not surprising, because only molecules with small moments of inertia *I* were included in the comparisons. The molecule with the highest molecular weight was toluene. Nevertheless, Ellis and Mayhew^31^ come to the reasonable conclusion that the theoretical values are just as reliable as the experimental values and that uncertainties of 20–30% must generally be expected. The *k*_cap_ values for the 114 target compounds of this work are listed in the ESI† (Appendix D) and were calculated for 353 K according to Su and Chesnavich.^29^ The parameterizations for determining *K*_cap_(*T*_R_) are also listed in the ESI.†

**Fig. 5:**
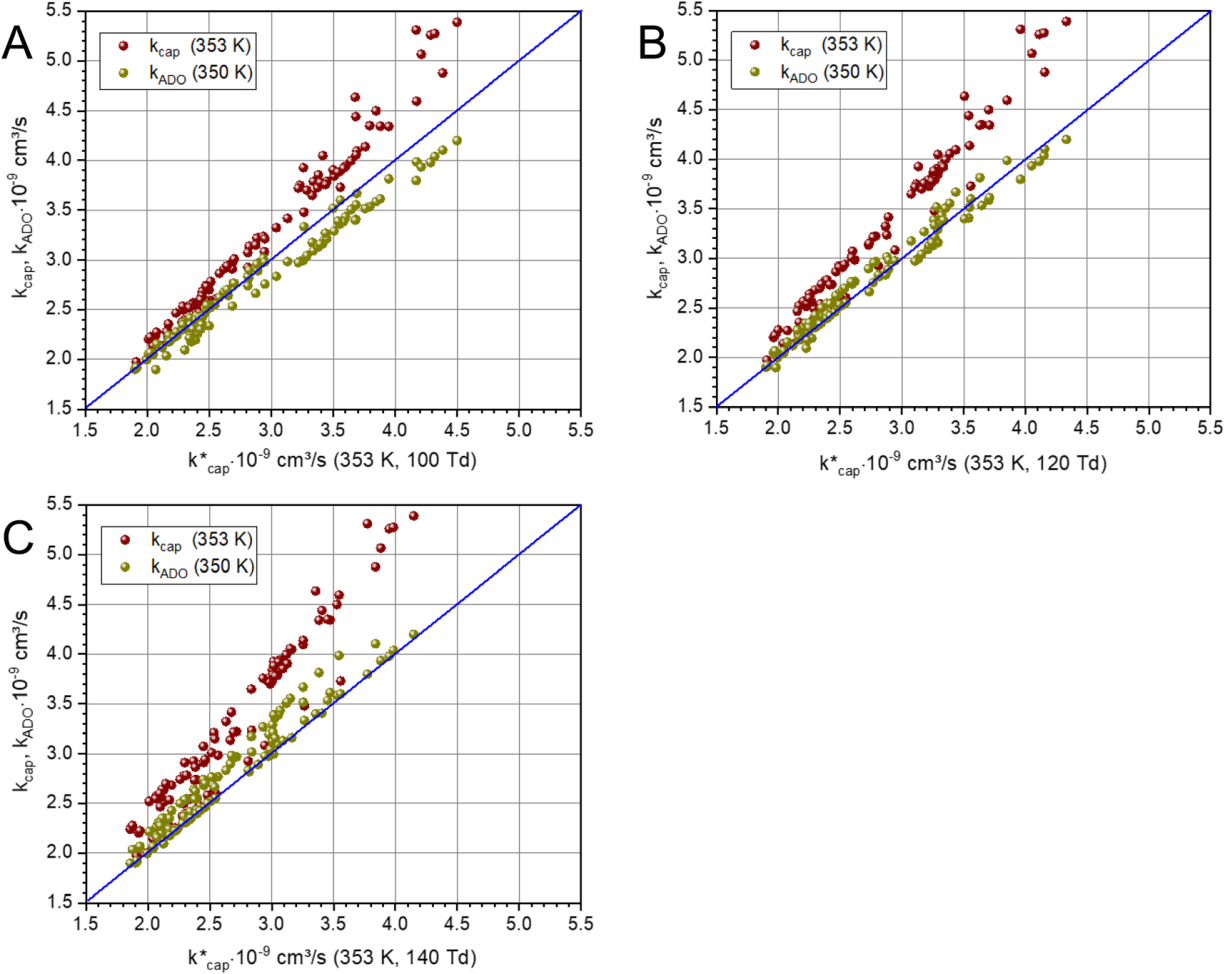
Plot of *k*_ADO_ and *k*_cap_*versus*
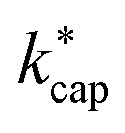
 at a temperature of 353 K (350 K for *k*_ADO_) for *E*/*N* values of 100 Td (A), 120 Td (B), and 140 Td (C). The data were taken from Table 3 and from the ESI.†

The ion–dipole collision theories used here have already been discussed by several authors,^9,10,31,50^ but essentially for small molecules. Tsikritea *et al.*^64^ state that the ADO theory and the capture theory according to Su and Chesnavich^29^ are appropriate when the rotational constants of the molecules are high. Calculated *k*_PT_ of larger molecules relevant for the indoor environment has only rarely been compared with experimental data, but the available results are satisfactory. Strictly speaking, on account of their symmetry, none of the molecules relevant to indoor analysis as well as those listed in Table 3 meet the preconditions of Su and Chesnavich.^29^ However, assuming the molecules to be linear rotors, the parameterization provided by Su^30^ is reasonable and the calculation of 
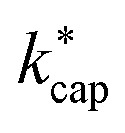
 should yield reliable results. The calculated values show the expected *E*/*N* dependent deviation from *k*_cap_ (see Fig. 5).

In general, one must be very careful and critical when comparing calculated and experimentally determined *k*_PT_ values. The rate constants depend not only on the temperature *T*_d_ in the reaction chamber, but also on the pressure *p*_d_ and in particular on the electric field in the drift tube, because these variables have a direct influence on the ion mobility. The advanced capture theory takes these aspects into account. The ion energies KE_ion_ required to calculate the 
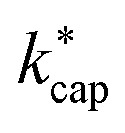
 values listed in Table 3 are based on the ion mobilities measured by Dotan *et al.*^43^ (see Table 1). The details of the parameterization for calculating 
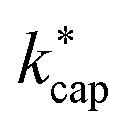
 from *K*_C_(*τ*, *ε*) can be found in the ESI.† In total, 
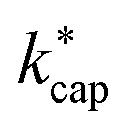
 was calculated for seven different *E*/*N* values between 80 Td and 140 Td at a temperature of 353 K. The advantages of Su's^30^ advanced capture theory over ADO theory and the original capture theory were discussed in detail by Cappellin *et al.*^50^ Using the example of seven sulfur compounds, it was also shown that the 
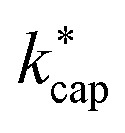
 values are larger than the *k*_ADO_ values. In a later work, Cappellin *et al.*^33^ measured *k*_PT_ values at *E*/*N* = 120 Td and *T*_d_ = 363 K for 11 substances that also play a role indoors. Excellent agreement with the calculated 
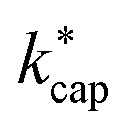
 values for these conditions was found. In all cases, the deviations between theory and experiment were less than 10%. Cappellin *et al.*^33^ also provide an extensive table of calculated 
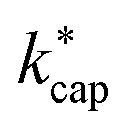
 values of organic compounds at 363 K as a function of *E*/*N*. Table 3 in this work essentially extends the list of Cappellin *et al.*^33^ with a large number of indoor-related compounds. In addition, as already mentioned, the applied quantum mechanical method for calculating dipole moments and polarizabilities is superior to previous approaches.

As far as our results are concerned, there is the expected good agreement with the data of Cappellin *et al.*^33^ The small deviations (≤5%) are due to the different data for dipole moment and polarizability as well as the different temperature (353 K *vs.* 363 K). The deviations in furfural are due to the fact that Cappellin *et al.*^33^ confused the dipole moments for the cis and trans isomer. The rate constants for glyoxal in Cappellin *et al.*^33^ are for the trans isomer and in our work for the cis isomer.

Of particular interest is the comparison of the different theories. For this purpose, *k*_ADO_ and *k*_cap_ are plotted *versus*
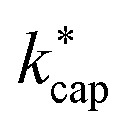
 in Fig. 5, for 100 Td, 120 Td and 140 Td, respectively. The blue line represents the 1 : 1 ratio. It can be seen that at 100 Td the *k*_ADO_ values are slightly below the 
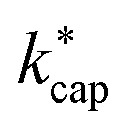
 values from about 3.0 × 10^−9^ cm^3^ s^−1^. At 120 Td the agreement between 
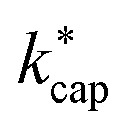
 and *k*_ADO_ is excellent, at 140 Td the *k*_ADO_ values deviate slightly upwards. Referring to the values shown in Fig. 5, the highest differences between 
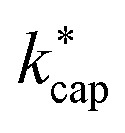
 and *k*_ADO_ for 100 Td and 140 Td are in the range of 10%, for 120 Td they are ≤5%. In this respect, for our assumed conditions and substances, we cannot confirm the statement by Ellis and Mayhew^31^ that ADO theory generally tends to underestimate the rate coefficients.

A different picture emerges for *k*_cap_. In the three cases displayed, the calculated *k*_cap_ values are well above the 1 : 1 line and show the expected systematic deviations from 
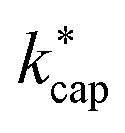
. However, in the case of *u*_0_ = constant, both *k*_cap_ and 
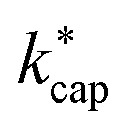
 do well agree within ≈±10% in the limit *E*/*N* → 0. Hence, the advanced trajectory analysis and its parametrization with a field dependent kinetic energy term has proven to be a sound and reasonable extension of the capture theory. In contrast to *k*_ADO_, the deviations of *k*_cap_ to 
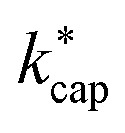
 increase with increasing rate constant and *E*/*N*. The highest deviations of about 30% occur at 140 Td with strongly polar substances such as acetonitrile, *N*-methyl-2-pyrrolidone and cis-furfural. In general, it is clear that deviations become more likely with increasing polarity and moment of inertia of the substance. For *μ*_D_ → 0 all theories merge into the Langevin equation. In addition, the capture theory according to Su and Chesnavich^29^ has methodological weaknesses when applied to larger molecules. In the calculation of the trajectories Chesnavich *et al.*^65^ have approximated all molecules as linear rotors irrespective of their effective symmetry. This means at least that the individual moments of inertia of the three mutually perpendicular axes of the molecule are averaged, which is a rough approximation for large molecules.

## 6 Conclusion

Proton transfer rate constants, which are necessary for the quantitative determination of airborne organic compounds in PTR-MS measurements, were calculated for 114 indoor air pollutants using ion–dipole collision theory at different levels. It was shown that this requires reliable dipole moments and polarizabilities, which are accessible for the respective ensemble containing the energetically lowest conformers in the gas phase *via* quantum mechanical calculations. The automation of these methods is necessary in order to be able to process a large number of molecules in a reasonable amount of time. In agreement with earlier work we conclude that the advanced capture theory according to Su^30^ provides the most reliable rate constants 
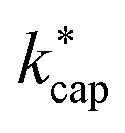
, since the conditions in the PTR-MS reaction chamber are taken into account with the energy of the ions KE_ion_, as is the *E*/*N* ratio. However, the ion mobilities in the reaction chamber as a function of *E*/*N* are required for the calculation.^3,43^ For *T*_d_ = 353 K and *E*/*N* = 120 Td, the ADO theory also provides reasonable values.

After analyzing the available experimental and theoretical data, we also agree with previous estimates that the rate constants can be determined with an accuracy of 10–25%. The uncertainties increase with the size of the molecule and increasing dipole moment. For more precise assessments, round-robin tests with certified gas standards are also required, which are not yet available for PTR-MS measurements.

## Author contributions

TS: conceptualization, methodology, investigation, formal analysis, visualization, writing – original draft; UH: methodology, investigation, formal analysis, verification, writing – original draft; MS: methodology, investigation, formal analysis, visualization, software, writing – original draft; SG: investigation, methodology, writing – review & editing.

## Conflicts of interest

The authors have no conflicts of interest to declare.

## Supplementary Material

RA-013-D3RA01705B-s001
