# Supplementary material for: Proton-transfer rate constants for the determination of organic indoor air pollutants by online mass spectrometry
Source: RSC Adv. 2023 Jun 13;13(26):17856–68. doi: 10.1039/d3ra01705b (PMC10262294; doi:10.1039/d3ra01705b)
Supplement: RA-013-D3RA01705B-s001 [file RA-013-D3RA01705B-s001.pdf]

Electronic Supplementary Material (ESI) for RSC Advances.
This journal is © The Royal Society of Chemistry 2023

SUPPORTING INFORMATION

Proton-transfer rate constants for the determination of organic indoor air pollutants by online mass spectrometry

Tunga Salthammer[1,*], Uwe Hohm[2], Marcel Stahn[3], Stefan Grimme[3]

1) Department of Material Analysis and Indoor Chemistry, Fraunhofer WKI, 38108 Braunschweig, Germany

2) Institute of Physical and Theoretical Chemistry, University of Braunschweig – Institute of Technology, 38106 Braunschweig, Germany

3) Mulliken Center for Theoretical Chemistry, Institute for Physical and Theoretical Chemistry, University of Bonn, 53115 Bonn, Germany

*) Corresponding author:

Prof. Dr. Tunga Salthammer

Email: tunga.salthammer@wki.fraunhofer.de

<u>Appendix A:</u> Calculation of the proton transfer reaction rate k_{ADO} from average dipole orientation (ADO) theory

Table S1. Temperature dependence of *C* according to Su and Bowers (1975) and Su et al. (1978).

**350 K (Su and Bowers, 1975)**		**300 K (Su et al., 1978)**	
$\mu_D/\alpha^{1/2}$	*C*	$\mu_D/\alpha^{1/2}$	*C*
0.000	0.000	0.0000	0.0000
0.163	0.039	0.1155	0.0913
0.250	0.076	0.2309	0.1416
0.327	0.106	0.3464	0.1740
0.408	0.130	0.4619	0.1962
0.490	0.149	0.5774	0.2122
0.572	0.164	0.6928	0.2241
0.653	0.178	0.8083	0.2332
0.735	0.189	0.9238	0.2403
0.817	0.198	1.1550	0.2506
0.898	0.206	1.2700	0.2542
0.980	0.213	1.3860	0.2573
1.061	0.220	1.5010	0.2591
1.143	0.225	1.6170	0.2615
1.225	0.230	1.7320	0.2632
1.306	0.234	1.8480	0.2645
1.388	0.238	1.9630	0.2656
1.470	0.242		
1.551	0.245		
1.633	0.248		
1.715	0.251		
1.796	0.253		
1.878	0.255		
1.960	0.257		
2.041	0.259		

Empirical equation (for best fit and interpolation purposes only:

$$C = A_1 + \frac{-A_1}{1+\left(\frac{\mu_D \cdot \alpha^{-1/2}}{A_2}\right)^p} \qquad \text{(S1)}$$

Non-linear regression analysis (Marquardt method), see Bevington and Robinson (2003), using ORIGIN 2021b (64-bit), see Table S2 for data.

Table S2. Best-fit parameters from equation (S1) for 300 K and 350 K.

**Temperature**	A_1	A_2	p
300 K	0.29366	0.24274	1.10725
350 K	0.28279	0.46805	1.56293

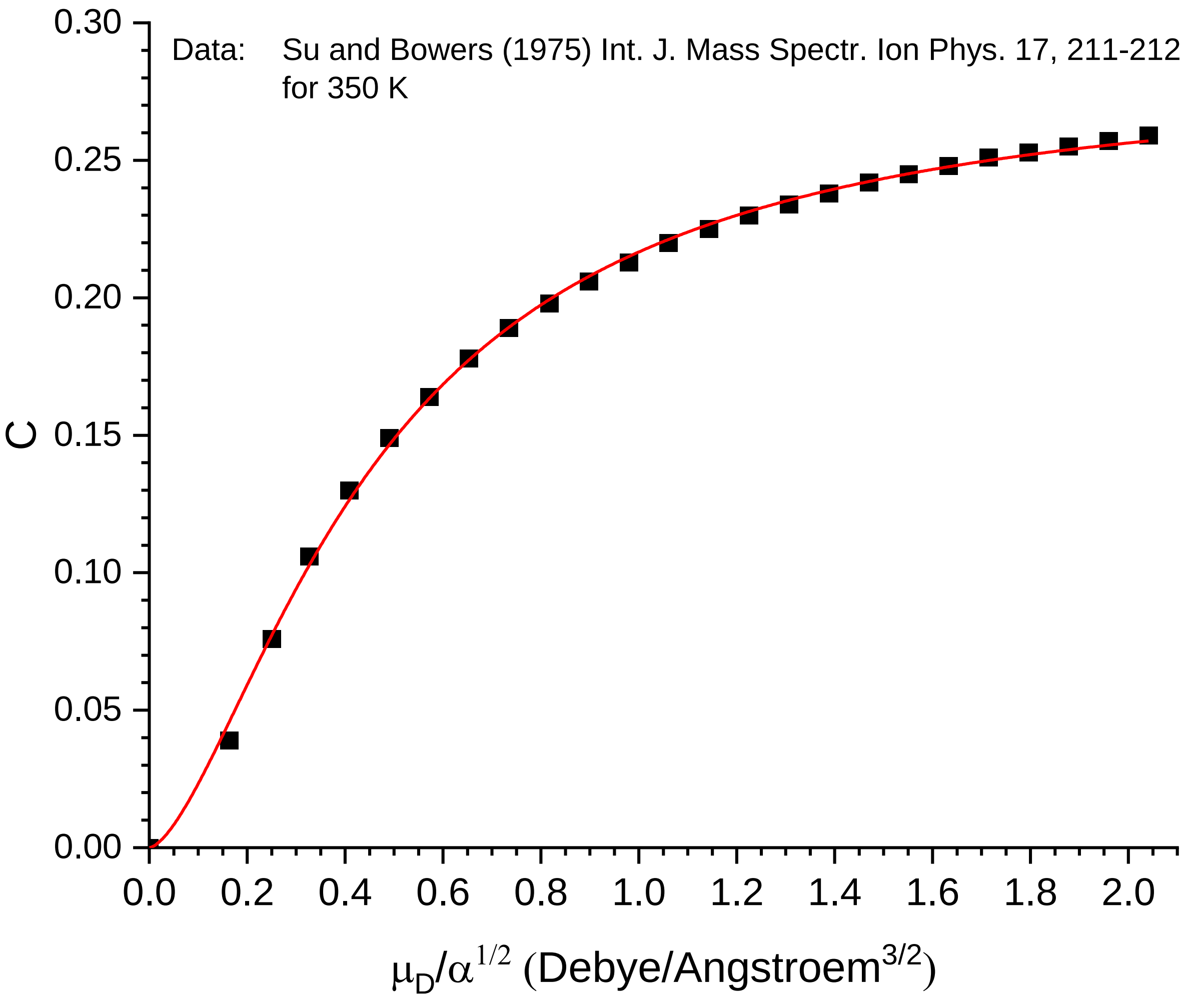

Figure S1. 350 K data from Table S1 and best-fit curve from equation S1. See Table S2 for fit parameters.

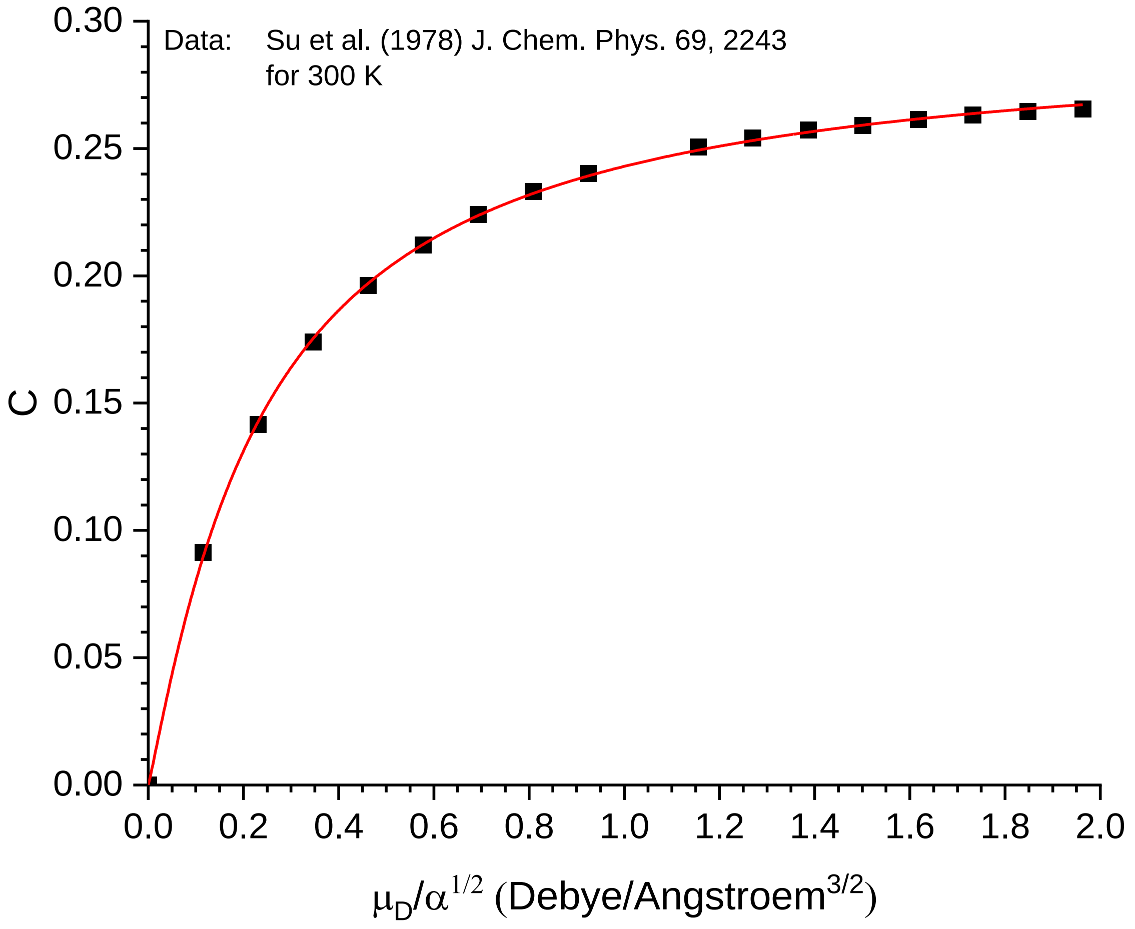

Figure S2. 300 K data from Table S1 and best-fit curve from equation S1. See Table S2 for fit parameters.

Calculation of k_{ADO} from ADO theory according to Su and Bowers (1973a, b)

<u>Note:</u> cgs units are used in the original work by Su and Bowers (1973a,b).

$$k_{ADO} = \frac{2 \cdot \pi \cdot q}{\sqrt{\mu_m}} \cdot \left[\sqrt{\alpha} + C \cdot \mu_D \cdot \sqrt{\frac{2}{\pi \cdot k_B \cdot T}} \right] \qquad \text{(S2)}$$

$$\frac{cm^3}{s} = \frac{\frac{\sqrt{g \cdot cm^3}}{s}}{\sqrt{g}} \left[\sqrt{cm^3} + \frac{\sqrt{g \cdot cm^3}}{s} \cdot cm \cdot \sqrt{\frac{s^2 \cdot K}{cm^2 \cdot g \cdot K}} \right] \qquad \text{(units for S2)}$$

$$\mu_m = \frac{MW_1 \cdot MW_2}{MW_1 + MW_2} \cdot \frac{1}{N_A} \tag{S3}$$

Symbol	Parameter	Unit
k_{ADO}	Proton-transfer reaction constant	$cm^3 \cdot s^{-1}$
q	Elementary charge	$4.802675108022 \cdot 10^{-10}$ Franklin
μ_m	Reduced mass	g
N_A	Avogadro Constant	$6.02214086 \cdot 10^{-23}$ mol^{-1}
α	Polarizability	$10^{-24} \cdot cm^3$
C	compensates for the effectiveness of the charge “locking in” the dipole	
μ_D	Dipole moment	Franklin·cm
k_B	Boltzmann Constant	$1.308064852 \cdot 10^{-16}$ $erg \cdot K^{-1}$
T	Temperature	Kelvin

1 Franklin (Fr) = $3.335641 \cdot 10^{-10}$ Coulomb (C)

$1.602 \cdot 10^{-19}$ C = $4.8026751080227 \cdot 10^{-10}$ Franklin

1 Fr = 1 $\frac{\sqrt{g \cdot cm^3}}{s}$

1 Debye = $1 \cdot 10^{-18}$ Fr·cm

$1.380649 \cdot 10^{-23}$ $J \cdot K^{-1}$ ($m^2 \cdot kg \cdot s^{-2} \cdot K^{-1}$) = $1.380649 \cdot 10^{-16}$ $erg \cdot K^{-1}$

1 erg = 1 $\frac{cm^2 \cdot g}{s^2}$

In SI units, equation (S2) reads as follows:

$$k_{ADO} = \sqrt{\frac{\pi \cdot \alpha \cdot q^2}{\varepsilon_0 \cdot \mu_m}} + \frac{C \cdot \mu_D \cdot q}{\varepsilon_0} \cdot \sqrt{\frac{1}{2 \cdot \pi \cdot \mu_m \cdot k_B \cdot T}} \tag{S2a}$$

Note that according to equation (2a) k_{ADO} is obtained in the unit m^3/s (for SI units of the parameters and constants see Appendix B).

1 cm^3/s = 10^{-6} m^3/s

Appendix B: Calculation of the proton transfer reaction rate k_{cap} from ion-polar molecule collision trajectory analysis according to Su and Chesnavich (1982).

$$k_L = \sqrt{\frac{\pi \cdot \alpha \cdot q^2}{\mu_m \cdot \epsilon_0}} \qquad \text{(S4)}$$

$$k_{cap} = k_L \cdot K_{cap}(T_R) \qquad \text{(S5)}$$

$$T_R = 4 \cdot \pi \cdot \varepsilon_0 \cdot \frac{2 \cdot \alpha \cdot k_B \cdot T}{\mu_D^2} \qquad \text{(S6)}$$

$$x = \sqrt{\frac{1}{T_R}} \qquad \text{(S7)}$$

K_{cap} (T_R)	0.9754 + (x + 0.509)²/10.526	x ≤ 2
	0.4767·x + 0.6200	2 < x ≤ 3
	0.5781·x + 0.3165	3 < x ≤ 35

K_{cap} is insensitive to the moment of inertia *I* when equation (S8) is met (Su and Chesnavich, 1982).

$$I^* = \frac{\mu_D \cdot I}{\alpha \cdot q \cdot \mu_m} < \frac{0.7 + x^2}{2 + 0.6 \cdot x} \qquad \text{(S8)}$$

**Symbol**	**Parameter**	**Unit**
k_{cap}	Proton-transfer reaction constant	$m^3 \cdot s^{-1}$
q	Elementary charge	$1.602 \cdot 10^{-19}$ C
ε_0	Vacuum permittivity	$8.854 \cdot 10^{-12}$ $F \cdot m^{-1}$
μ_m	Reduced mass	kg
N_A	Avogadro Constant	$6.02214086 \cdot 10^{-23}$ mol^{-1}
α	Polarizability	m^3
μ_D	Dipole moment	C·m
k_B	Boltzmann Constant	$1.380649 \cdot 10^{-23}$ $J \cdot K^{-1}$
T	Temperature	Kelvin

<u>Appendix C:</u> Calculation of the proton transfer reaction rate k^{*}_{cap} from advanced ion-polar molecule collision trajectory analysis according to Su (1994).

$$k^{*}_{cap} = k_L \cdot K_C(\tau, \varepsilon) \tag{S9}$$

$$\tau = \frac{\mu_D}{\sqrt{\alpha \cdot T}} \tag{S10}$$

$$\varepsilon = \frac{\mu_D}{\sqrt{\alpha \cdot KE_{CM}}} \tag{S11}$$

$$K_C(\tau, \varepsilon) = 1 + C_1 \cdot \tau^{0.4} \cdot \varepsilon^2 \cdot S + C_2 \cdot (1 - S) \cdot sin[C_3 \cdot \{C_4 + \ln(\tau)\}] \cdot \tau^{0.6} \cdot (\varepsilon - 0.5)^{1/2} \tag{S12}$$

C_1 = 0.727143

C_2 = 3.71823

C_3 = 0.586920

C_4 = 4.97894

S	exp[-2·(ε - 1.5)]	$\varepsilon > 1.5$
	1	$\varepsilon \leq 1.5$

Symbol	Parameter	Unit
k_L	Langevin constant (see eq. S4)	$cm^3 \cdot s^{-1}$<br>(convert from $m \cdot s^{-1}$ if necessary)
k^{*}_{cap}	Proton-transfer reaction constant	$cm^3 \cdot s^{-1}$
α	Polarizability	Å^3 (10^{-24} cm^3)
μ_D	Dipole moment	Debye
T	Temperature	Kelvin
KE_{cm}	Center of mass kinetic energy	eV

$$KE_{ion} = \frac{3}{2} \cdot k_B \cdot T + \frac{1}{2} \cdot m_{ion} \cdot {v_d}^2 + \frac{1}{2} \cdot m_{air} \cdot {v_d}^2 \quad \text{(S13)}$$

$$KE_{CM} = \frac{m_m}{m_m + m_{ion}} \left(KE_{ion} - \frac{3}{2} \cdot k_B \cdot T \right) + \frac{3}{2} \cdot k_B \cdot T \quad \text{(S14)}$$

**Symbol**	**Parameter**	**Unit**
m_{ion}	mass of the respective ion (H_3O^+ or $H_3O^+ \cdot n(H_2O)$)	kg
m_{air}	mass of the buffer gas (MW_{air} (dry) = 28.96 $g \cdot mol^{-1}$)	kg
m_m	mass of the molecule	kg
v_d	Ion velocity in the drift tube	$m \cdot s^{-1}$
k_B	Boltzmann constant	$1.380649 \cdot 10^{-23}$ $J \cdot K^{-1}$
T	absolute temperature	Kelvin
KE_{ion}	Total mean ion kinetic energy	J
KE_{CM}	Center of mass kinetic energy	J

Note: KE_{ion} and KE_{CM} are obtained in Joule. KE_{CM} must be converted to eV before used in equation (S11).

1 eV = $1.602 \cdot 10^{-19}$ J

Appendix D: Compounds and parameters

Table S3. Monoisotopic molecular masses (MW) and calculated rate constants k_{cap} and k_{ADO}.

Compound	CAS	MW [1] (g/mol)	k_{cap} (10^{-9} cm^3/s)	k_{ADO} (10^{-9} cm^3/s)
**Aliphatic and cyclic hydrocarbons**				
n-Hexane	110-54-3	86.11	2.00	1.99
n-Heptane	142-82-5	100.13	2.13	2.12
n-Octane	111-65-9	114.14	2.25	2.24
n-Nonane	111-84-2	128.16	2.36	2.35
n-Decane	124-18-5	142.17	2.47	2.46
Cyclohexane	110-82-7	84.09	1.91	1.91
Methylcyclohexane	108-87-2	98.11	2.07	2.05
4-Vinyl cyclohexene (4-VCH)	100-40-3	108.09	2.23	2.18
**Aromatic hydrocarbons**				
Benzene	71-43-2	78.05	1.90	1.90
Toluene	108-88-3	92.06	2.14	2.07
Ethylbenzene	100-41-4	106.08	2.27	2.19
o-Xylene	95-47-6	106.08	2.36	2.24
m-Xylene	108-38-3	106.08	2.27	2.20
p-Xylene	106-42-3	106.08	2.18	2.18
1,2,3-Trimethylbenzene	526-73-8	120.09	2.50	2.37
1,2,4-Trimethylbenzene	95-63-6	120.09	2.39	2.32
1,3,5-Trimethylbenzene	108-67-8	120.09	2.31	2.31
Isopropylbenzene	98-82-8	120.09	2.37	2.30
Styrene	100-42-5	104.06	2.25	2.22
Chlorobenzene	108-90-7	112.01	2.93	2.64
1,2-Dichlorobenzene	95-50-1	145.97	3.65	3.17
1,4-Dichlorobenzene	106-46-7	145.97	2.18	2.18
4-Phenyl cyclohexene (4-PCH)	4994-16-5	158.11	2.60	2.55
**Polycyclic aromatic hydrocarbons**				
Naphthalene	91-20-3	128.06	2.40	2.40
1-Methylnaphthalene	90-12-0	142.08	2.59	2.52
1-Chloronaphthalene	90-13-1	162.02	3.22	2.97
Anthacene	120-12-7	178.08	2.89	2.89

Phenanthrene	85-01-8	178.08	2.82	2.82
**Terpenoids**				
Isoprene	78-79-5	68.06	1.97	1.92
α-Pinene	80-56-8	136.13	2.37	2.33
β-Pinene	127-91-3	136.13	2.54	2.42
3-Carene	13466-78-9	136.13	2.38	2.35
d-Limonene	5989-27-5	136.13	2.54	2.44
α-Phellandrene	4221-98-1	136.13	2.44	2.40
Myrcene	123-35-3	136.13	2.58	2.50
β-Caryophyllene	87-44-5	204.19	2.93	2.83
Terpinolene	586-62-9	136.13	2.47	2.42
Linalool	78-70-6	154.14	3.14	2.90
α-Terpineol	98-55-5	154.14	2.99	2.77
**Alcohols**				
Methanol	67-56-1	32.03	2.51	2.09
Ethanol	64-17-5	46.04	2.56	2.20
1-Propanol	71-23-8	60.06	2.56	2.25
2-Propanol	67-63-0	60.06	2.58	2.26
1-Butanol	71-36-3	74.07	2.63	2.35
2-Butanol	75-65-0	74.07	2.64	2.35
2-Methyl-1-propanol	78-83-1	74.07	2.57	2.31
1-Pentanol	71-41-0	88.09	2.68	2.43
1-Hexanol	111-27-3	102.10	2.78	2.53
1-Heptanol	111-70-6	116.12	2.87	2.62
2-Ethyl-1-hexanol	104-76-7	130.14	2.91	2.68
Benzyl alcohol	100-51-6	108.06	2.78	2.55
**Aldehydes**				
Acetaldehyde	75-07-0	44.03	3.93	3.00
Propanal	123-38-6	58.04	3.75	2.99
Butanal	123-72-8	72.06	3.70	3.04
2-Methylpropanal	78-84-2	72.06	3.79	3.09
Pentanal	110-62-3	86.07	3.73	3.13
Hexanal	66-25-1	100.09	3.76	3.19
Heptanal	111-71-7	114.10	3.84	3.29
Octanal	124-13-0	128.12	3.89	3.36
Nonanal	124-19-6	142.13	3.94	3.43

Decanal	112-31-2	156.15	4.00	3.51
Acrolein (trans)	107-02-8	56.03	4.44	3.41
Acrolein (cis)	107-02-8	56.03	3.72	2.97
trans-2-Butenal	123-73-9	70.04	5.26	3.98
trans-2-Hexenal	6728-26-3	98.07	5.39	4.20
Furfural (trans)	98-01-1	96.02	4.50	3.59
Furfural (cis)	98-01-1	96.02	5.27	4.04
Glyoxal (cis)	107-22-2	58.01	4.64	3.40
Benzaldehyde	100-52-7	106.04	4.35	3.61
**Ketones**				
Acetone	67-64-1	58.04	4.05	3.16
2-Butanone (MEK)	78-93-3	72.06	3.85	3.13
4-Methyl-2-pentanone (MIBK)	108-10-1	100.09	3.79	3.21
Cyclohexanone	108-94-1	98.07	4.35	3.53
6-Methyl-5-heptene-2-one (6-MHO)	110-93-0	126.10	3.92	3.38
4-Oxopentanal (4-OPA)	626-96-0	100.05	3.78	3.16
Geranylacetone	3796-70-1	194.17	4.10	3.67
3-Octanone	106-68-3	128.12	3.76	3.27
**Esters**				
Methyl acetate	79-20-9	74.04	2.70	2.34
Ethyl acetate	141-78-6	88.05	2.91	2.54
n-Butyl acetate	123-86-4	116.08	3.07	2.74
2-Ethylhexyl acetate	103-09-3	172.15	3.22	2.96
Dimethyl phthalate	131-11-3	194.06	4.06	3.55
Diethylphthalate	84-66-2	222.09	3.90	3.52
Dimethyl succinate	106-65-0	146.06	2.53	2.35
Dimethyl adipate	627-93-0	174.09	2.74	2.55
Dimethyl sebacate	106-79-6	230.15	3.23	3.02
**Glycols**				
2-Ethoxyethanol	110-80-5	90.07	3.32	2.83
1-Methoxy-2-propanol	107-98-2	90.07	3.21	2.76
1,2-Propanediol	57-55-6	76.05	3.15	2.66
2-Butoxyethanol	111-76-2	118.10	3.42	2.98
2-Butoxyethoxyethanol	54446-78-5	162.13	3.88	3.39
Phenoxyethanol	122-99-6	138.07	2.94	2.70
2-Butoxyethyl acetate	112-07-2	160.11	3.01	2.77

**Acids**				
Formic acid	64-18-6	46.01	2.24	1.90
Acetic acid	64-19-7	60.02	2.55	2.18
Propionic acid	79-09-4	74.04	2.52	2.21
Hexanoic acid	142-62-1	116.08	2.74	2.50
Isobutyric acid	79-31-2	88.05	2.64	2.35
**Phenones**				
Acetophenone	98-86-2	120.06	4.14	3.51
Benzophenone	119-61-9	182.07	4.34	3.81
Darocur 1173	7473-98-5	164.08	4.88	4.10
Irgacure 184	947-19-3	204.12	4.60	3.99
**Siloxanes**				
D4	556-67-2	296.08	3.08	2.98
D5	541-02-6	370.09	3.48	3.33
D6	540-97-6	444.11	3.73	3.60
**Other**				
Acetonitrile	75-05-8	41.03	5.31	3.80
Ethylamine	75-04-7	45.06	2.28	2.03
Diethylamine	109-89-7	73.09	2.20	2.05
Triethylamine	121-44-8	101.12	2.27	2.16
Dimethyl sulfide	75-18-3	62.02	2.60	2.30
1,3-Benzothiazol	95-16-9	135.01	2.73	2.54
N-Methyl-2-pyrrolidon	872-50-4	99.07	5.07	3.93
2-Butanonoxim	96-29-7	87.07	2.22	2.07
Phenol	108-95-2	94.04	2.47	2.28

1) calculated for the most abundant isotopes

References

Bevington, P.R., Robinson, D.K., 2003. Data reduction and error analysis for the physical sciences. McGraw Hill, New York.

Su, T., 1994. Parametrization of kinetic energy dependences of ion–polar molecule collision rate constants by trajectory calculations. Journal of Chemical Physics 100, 4703-4703.

Su, T., Bowers, M.T., 1973a. Theory of ion-polar molecule collisions. Comparison with experimental charge transfer reactions of rare gas ions to geometric isomers of difluorobenzene and dichloroethylene. Journal of Chemical Physics 58, 3027-3037.

Su, T., Bowers, M.T., 1973b. Ion-polar molecule collisions: the effect of ion size on ion-polar molecule rate constants; the parameterization of the average-dipole-orientation theory. International Journal of Mass Spectrometry and Ion Physics 12, 347-356.

Su, T., Bowers, M.T., 1975. Parameterization of the average dipole orientation theory: temperature dependence. International Journal of Mass Spectrometry and Ion Physics 17, 211-212.

Su, T., Chesnavich, W.J., 1982. Parametrization of the ion–polar molecule collision rate constant by trajectory calculations. Journal of Chemical Physics 76, 5183-5185.

Su, T., Su, E.C.F., Bowers, M.T., 1978. Ion–polar molecule collisions. Conservation of angular momentum in the average dipole orientation theory. The AADO theory. Journal of Chemical Physics 69, 2243-2250.
